# Interments in the City and Liberties of Philadelphia, from the 4th to the 11th of December

**Published:** 1841-12-18

**Authors:** 


### HEALTH OF THE CITY.

INTERMENTS *in the City and Liberties of Philadelphia, from the 4th to the 11th of December.*

| Diseases. | Adults. | Children. |
|---|---|---|
| Asthma, | 1 | 0 |
| Apoplexy | 1 | 0 |
| Burns, | 0 | 1 |
| Casualty, | 1 | 0 |
| Croup, | 0 | 2 |
| Compound fracture of Skull, | 1 | 0 |
| Consumption of the lungs, | 11 | 1 |
| Convulsions, | 1 | 6 |
| Constipation, | 1 | 0 |
| Diarrhœa, | 1 | 0 |
| Dropsy, | 2 | 0 |
| —— Head, | 0 | 2 |
| —— Breast, | 0 | 1 |
| Disease of the Brain, | 3 | 1 |
| Dysentery, | 3 | 1 |
| Debility | 0 | 1 |
| Effusion on brain, | 0 | 1 |
| Fever, | 0 | 1 |
| —— Congestive, | 1 | 0 |
| —— Typhus, | 1 | 0 |
| —— Typhoid, | 2 | 0 |
| —— Scarlet, | 0 | 4 |
| Gangrene of lungs, | 1 | 0 |
| Inflammation of the Brain, | 1 | 1 |
| —— Bronchi, | 0 | 3 |
| —— Lungs, | 6 | 1 |
| —— Stomach, | 0 | 1 |
| —— Bowels, | 0 | 2 |
| —— Liver, | 1 | 0 |
| | — | — |
| *Carried forward,* | 36 | 36 |
| *Brought forward,* | 36 | 36 |
| Inflammation of the Peritonæum, | 0 | 1 |
| Inanition, | 0 | 1 |
| Marasmus, | 1 | 2 |
| Mania a Potu, | 1 | 0 |
| Neglect and exposure, | 1 | 0 |
| Old age, | 6 | 0 |
| Paralysis of the Lungs, | 1 | 0 |
| Pleurisy, | 1 | 0 |
| Small pox, | 2 | 5 |
| Still-born, | 0 | 5 |
| Tabes Mesenterica, | 0 | 1 |
| | — | — |
| Total, 97— | 52 | 45 |

Of the above, there were under 1 year, 28

| | |
|---|---|
| From 1 to 2 | 6 |
| 2 to 5 | 5 |
| 5 to 10 | 4 |
| 10 to 15 | 0 |
| 15 to 20 | 2 |
| 20 to 30 | 13 |
| 30 to 40 | 10 |
| 40 to 50 | 7 |
| 50 to 60 | 6 |
| 60 to 70 | 5 |
| 70 to 80 | 5 |
| 80 to 90 | 5 |
| 90 to 100 | 1 |
| Total, | 97 |

Of the above there were 9 from the almshouse, and 12 people of colour, which are included in the total amount.

